# Comparison of measured and computed portal dose for IMRT treatment

**DOI:** 10.1120/jacmp.v7i3.2281

**Published:** 2006-08-24

**Authors:** Savino Cilla, Pietro Viola, Luigi Azario, Luca Grimaldi, Maurizio Craus, Guido D'Onofrio, Andrea Fidanzio, Francesco Deodato, Gabriella Macchia, Cinzia Digesù, Alessio G. Morganti, Angelo Piermattei

**Affiliations:** ^1^ U.O. Fisica Sanitaria Centro di Ricerca e Formazione ad Alta Tecnologia nelle Scienze Biomediche Università Cattolica S. Cuore Campobasso Italy; ^2^ U.O. Radioterapia Centro di Ricerca e Formazione ad Alta Tecnologia nelle Scienze Biomediche Università Cattolica S. Cuore Campobasso Italy; ^3^ Istituto di Fisica Università Cattolica S. Cuore Roma Italy

**Keywords:** portal dose, IMRT, radiotherapy

## Abstract

A new 2D array Seven 29™ model (PTW, Freiburg), equipped with 729 vented plane‐parallel ion chambers, projected for pretreatment verification of radiotherapy plans, was used as a detector for the transmitted or portal dose measurements below a Rando phantom. The dosimetric qualities of the 2D array make it attractive for measuring transmitted dose maps from step‐and‐shoot intensity‐modulated radiotherapy (IMRT). It is well known that for step‐and‐shoot IMRT beams that use a small number of monitor units (MUs) per sequence, the early and recent electronic portal imaging devices (EPIDs) present a different response at X‐ray start‐up that affects the accuracy of the measured transmitted dose. The comparison of portal doses measured to those calculated by a commercial treatment‐planning system (TPS) can verify correct dose delivery during treatment. This direct validation was tested by irradiating a simulated head tumor in a Rando anthropomorphic phantom by step‐and‐shoot IMRT beams. The absolute transmitted doses on a plane orthogonal to the beam central axis below the phantom were measured by the 2D array calibrated in terms of dose to water and compared with the computed portal dose extracted by custom software. In a previous paper, the comparison between the IMRT portal doses, computed by a commercial TPS and measured by a linear array that supplied a 1 mm spatial dose resolution, was carried out. The γ‐index analysis supplied an agreement of more than 95% of the dose point with acceptance criteria, in terms of dose difference, $\Delta D_{\max}$, and distance agreement, $\Delta d_{\max}$, equal to 4% and 4 mm, respectively. In this paper, we verify the possible use of the PTW 2D array for measurements of the transmitted doses during several fractions of head and neck tumor radiotherapy. There are two advantages in the use of this 2D array as a portal dose device for the IMRT quality assurance program: first is the ability to perform absolute dose comparisons for hundreds of measurement positions to verify the correct dose delivery in several fractions of the therapy; second is the efficiency in time to detect these kinds of dose distributions within the field of view area of the CT scanner.

PACS number: 87.53.Xd

## I. INTRODUCTION

Inverse planned intensity‐modulated radiotherapy (IMRT) treatment is capable of producing complex dose distributions that can conform to even a concave volume. However, because of the production of steep dose gradients associated with IMRT, the *in vivo* verification of delivered dose in several fractions of the therapy is prudent for the assurance of patient safety.

Although sophisticated means to calculate and deliver modulated dose distributions have been developed, means to verify their actual delivery by radiographic film, 2D arrays, and electronic portal imaging devices (EPIDs) are often relatively cumbersome. In addition, these detectors are generally used for standard tests in pretreatment conditions to verify the dose map in homogeneous standard phantoms.

The EPID seems to be a valuable tool for *in vivo* quality assurance purposes,^(^ ^1^ ^–^ ^4^ ^)^ in particular, to assure beam centering by visual inspection. However, there does not exist any direct and practical method to check the correct positioning of the patient, the correct leaf position, and the dosimetry during treatment. In some institutions, methods to determine the transmitted or portal dose distribution below the patient have been developed to obtain a comparison between planned and reconstructed dose distributions in patient.^(^ ^5^ ^–^ ^7^ ^)^ Indeed, if accurate electron density information in the CT scanners truly represents the patient in the treatment position, and the dose calculation of the treatment‐planning system (TPS) below the patient is accurate, it is possible to compare computed and measured online portal doses within the field of view (FOV) of the CT, to detect incorrect dose delivery or changes in the centering of the patient. Unfortunately, early generation liquid‐filled matrix ion chambers (LiFi) and the camera‐based fluoroscopic EPID generally produced images with low contrast and limited stability, the latter due to temperature fluctuations and radiation damage. Moreover, their nonlinear dose response and field‐size‐dependent spreading of optical photons make them difficult to calibrate and use clinically. A third and more recent class of EPIDs uses amorphous silicon and is more efficient.^(^ ^4^ ^,^ ^8^ ^)^

The limitations of the EPIDs used as detectors for X‐ray transmitted dosimetry were analyzed, and complex solutions have been proposed to take into account the following:

- duty cycle (how much of the “beam‐on” time is actually integrated by the imager)
- sensitivity (how large the noise is on the final imager)
- stability (how much the detector gain varies with time, temperature, and gantry angle)
- dose response (relationship between camera signal and dose to water)

Moreover, for step‐and‐shoot IMRT applications, many beam sequences with a small number of monitor units (MUs) are often used, and the deviations occurring at beam start‐up significantly affect the accuracy of the EPID portal dosimetry.^(^ ^2^ ^,^ ^9^ ^)^ In other words, the EPID phantom, having dose‐deposition properties that differ significantly from those of a simple water phantom (which could be easily simulated by a TPS), presents a complicated dose–response relationship.

Another dosimetric device that presents high spatial resolution is radiographic film. Radiographic film requires many hours of work and extreme care in film processing. Indeed, generally the films are oversensitive to low‐energy photons, which may be important in penumbra regions. Moreover, due to the absence of chemical processing of radiographic films in our center (as in many other hospital centers), film dosimetry was not considered.

Recently, the authors performed measurements of portal doses for square fields on the Rando phantom by a calibrated linear (1D) array, LA48 PTW,^(10)^ that uses 47 liquid ion chambers^(11)^ that can be moved in a plane to obtain dose distributions with a spatial resolution of 1 mm. These measurements were compared with the computed portal dose profiles obtained by three different TPSs, using Low et al.'s analysis.^(12)^ Generally, TPSs do not supply accurate absolute or relative dose calculations outside the body contours and, in particular, at the distance where the EPID operates. The TPS Plato version Sunrise supplied the best results^(10)^ in terms of agreement between computed and measured absolute portal dose profiles on a plane orthogonal to the beam central axis within the FOV area of the CT scanner. On the basis of these results, the acceptance criteria for the Plato portal dose computation for head and neck tumors irradiated by step‐and‐shoot IMRT were determined again using LA48 measured profiles.

A 2D array (PTW, Freiburg, Germany), consisting of 729 air ion chambers, was used in this work as a device for direct and online measurement of transmitted dose on a plane. The 2D array can feasibly verify accurate dose delivery of complex fluence modulated fields in a standard plastic water‐equivalent phantom for pretreatment quality control. Recently, the suitability of the PTW 2D array was analyzed^(13)^ in terms of reproducibility in the short, medium, and long term. Moreover, the array successfully detects positional movements at the millimeter scale of the LINAC secondary collimators and presents good water equivalence (which can be easily simulated in the TPS). The measurements reported in this paper are encouraging for use as a device for measuring transmitted doses below a Rando head phantom during the IMRT treatment, to compare with the TPS computation.

## II. MATERIALS AND METHODS

### A. LINAC

A 6‐MV X‐ray beam, delivered by an Elekta Precise LINAC, was used in this work. The 6‐MV X‐ray beam generally operates for radiotherapy treatments with a nominal $330\;\text{MU}\;\min^{-1}$, corresponding to $3.3\;\text{Gy}\;\min^{-1}$ at the reference depth of 10 cm for a $10\times 10\;{\text{cm}}^{2}$ field size at a phantom source‐to‐surface distance (SSD) of 100 cm. The Elekta Precise LINAC is equipped with a standard multileaf collimator (MLC), which consists of two leaf banks of 40 leaves each, is 1 cm wide at the isocenter distance of 100 cm, and can operate in step‐and‐shoot mode for IMRT.

The portal imager of the Elekta Precise is an early generation camera‐based fluoroscopic EPID, and for the reasons reported in the introduction, it was not used as a portal dose detector.

### B. PTW 2D array

The PTW 2D array model L981359 is equipped with 729 vented plane‐parallel ion chambers with a $0.6\;g/{\text{cm}}^{2}$ graphite wall. Every ion chamber, $5\times 5\;{\text{mm}}^{2}$ surface area and 5 mm thick, is polarized with 400 V; the reference point is located 0.5 cm from the 2D array surface. Spaced 1 cm apart, the chambers are located in a 27×27 matrix. The 2D array external dimensions are $30\times 42\times 2.2\;{\text{cm}}^{3}$, and the surrounding material is polymethyl methacrylate (PMMA). The measuring system (mass 2.4 kg) consists of the chamber array itself, which also accommodates part of the electronic device, the array interface, and a data acquisition board for the personal computer. As specified by the manufacturer, the 2D array supplies dose measurements with high reproducibility in a range between 0.2 Gy and 10 Gy and dose rates in a range between $0.5\;\text{Gy}\;\min^{-1}$ and $8\;\text{Gy}\;\min^{-1}$, with a resolution of $1\;\text{mGy}\;\min^{-1}$ (using a display cycle that can be selected from between 400 ms and 999 ms).

The dosimetric characterization of the 2D array was recently published.^(13)^ The extensive measurements performed in that work show that the 2D array is reproducible in the short, medium, and long term, and it presents a good output factor response for small radiation fields. However, the most important characteristic is that the array is very sensitive to positional changes on the millimeter scale of the collimation system. The limitations of the array‐sampling capabilities due to the detector design with a 1‐cm spacing of ion chambers was analyzed, comparing dose profiles with high‐dose gradient regions, obtained with a pinpoint ion chamber. Nonetheless, the agreement between the two datasets was very good. In other words, the 2D array data accurately matched the reference ion chamber profiles when the collimation system was moved 1 mm at a time.

In this work, the dosimetric accuracy of the 2D array was confirmed using a linear array, LA48 PTW. This device contains 47 liquid ion chambers (spaced 8 mm apart, $4\times 4\;{\text{mm}}^{2}$ in size, and 0.5 mm thick), and it can supply dose distributions with a spatial resolution of 1 mm. Martens et al.^(11)^ and the present authors^(10)^ observed that the dose profiles supplied by the LA48 presented differences with the profiles obtained with a PTW natural diamond detector (type 60003) well within a $\Delta D_{\max}$ of 1.5% and a $\Delta d_{\max}$ of 1 mm for square fields of 2 cm, 5 cm, 10 cm, 15 cm, and 20 cm side length and intensity‐modulated (IM) beams. For these measurements the diamond detector ($3\times 3\;{\text{mm}}^{2}$ in size and 0.27 mm thick) was oriented for the maximum spatial resolution in the scan direction. As mentioned above, in this work the comparison between the dose profiles of open and wedge fields obtained by the LA48 in water phantom and those measured by the 2D array in PMMA phantom was carried out using custom software called Cilgam, described in section C and developed to verify the agreement between computed and measured doses. The dose profiles were measured for different square fields, 3 cm, 5 cm, 15 cm, 20 cm, and 25 cm side length. Some comparisons were carried out for the IM beams used in this work for the head and neck tumor.

The commissioning of the 2D array in a PMMA phantom was examined in this work to verify the signal reproducibility for doses as low as 0.01 Gy, with the aim of using the device for transmitted absolute dose measurements. A PTW thimble ion chamber type (model TM31002; active volume 0.125 cm^3^ air) was used as a reference detector for the absolute dose measurements in a water phantom, the latter carried out using the practical code IAEA TRS 398^(14)^ to determine the absolute dose values.

**Figure 1:**
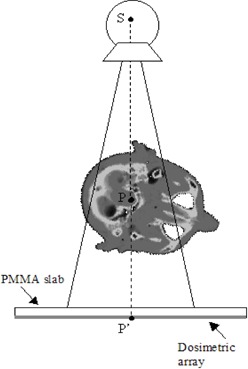
Experimental setup of a Rando head phantom for the simulation of an IMRT beam at 252°. A PMMA slab is placed over the dosimetric array. In a previous work,^10^) the dosimetric array was an LA48 linear array; in this work, it is a 2D array. The distance of the isocenter, *P*, from the source, *S*, is 100 cm; the distance *PP'* is 20 cm.

When the 2D array was used as a portal dose detector, the 2D array was positioned at the depth of maximum dose (buildup) for the Elekta 6‐MV X‐ray beam (1.55 cm of water equivalent material), obtained with an adjunctive PMMA 0.8 cm slab (Fig. 1).

The signal, *M*, of the 2D array central ion chamber was at first corrected for standard pressure and temperature, and calibrated in terms of dose to water using a $10\times 10\;{\text{cm}}^{2}$ field size at SSD=100 cm and $330\;\text{MU}\;\min^{-1}$. A range of dose values between 1 cGy and 10 Gy was used.

The dose rate dependence of the 2D array central ion chamber was analyzed measuring dose rates between $0.1\;\text{Gy}\;\min^{-1}$ and $5\;\text{Gy}\;\min^{-1}$, obtained by changing the source detector distance and the MU rate. In this range the correction factor for the ion recombination of the PTW reference ion chamber ranged between 1.001 and 1.002. These measurements were performed with the PMMA buildup slab on the top of the 2D array.

The manufacturer of the 2D array supplied normalization factors for each chamber relative to the central ion chamber. These factors were verified by irradiating the array with a $30\times 30\;{\text{cm}}^{2}$ field and comparing dose profiles extracted from the array to those measured by the thimble chamber.

Using an SSD of 100 cm, the signal of the central ion chamber was analyzed to obtain the total scatter correction factor $\left(S_{\text{cp}}\right)$ at the buildup using square fields, between $2\times 2\;{\text{cm}}^{2}$ and $20\times 20\;{\text{cm}}^{2}$. This measurement was carried out to verify the presence of the fluence perturbation due to the neighboring ion chambers as a function of field size.

Generally, the dose calibration in terms of centigrays per monitor unit is done for a number equal to or greater than 100 MUs, once the beam has stabilized. Due to the low number of MUs used for the sequences of a step‐and‐shoot IMRT beam, a set of measurements with the central ion chamber of the 2D array was carried out at buildup. A range of MUs between 1 and 100 with $330\;\text{MU}\;\min^{-1}$ and beam of $10\times 10\;{\text{cm}}^{2}$ at phantom surface was used to check the stability of the dose per MU of the Elekta Precise LINAC.

PTW software called VeriSoft was used to convert 2D dose maps from the TPS to gray scale virtual films. These virtual films can be presented on the screen of a personal computer with the locations of the 729 ion chambers superimposed over the gray scale image.

In this work, we investigated the feasibility of the PTW 2D array to detect some delivery errors, simulating ±5 mm shifts of the phantom isocenter or rotating the beam collimators of 5°.

### C. γ‐Index analysis

Custom software, called Cilgam, was implemented to verify the agreement between the profiles measured and computed by the TPS Plato Sunrise. Following the Low et al. analysis,^(12)^ all computed dose values on a “calculation plane” were converted to percent values by dividing them by the maximum dose measured on the same plane.^(15)^ Quantitative comparisons between the measured and calculated dose at the vectorial spatial location ${\vec{r}}_{m}$ and ${\vec{r}}_{c}$, in both the dose and the physical distance, scaled as a function of the acceptance criteria, were carried out using the γ‐index defined by the following expression(^12^):

(1) $$\gamma =\text{min}\left\{\sqrt{\frac{r^{2}({\vec{r}}_{m},{\vec{r}}_{c})}{\Delta d_{\text{max}}^{2}}+\frac{\delta^{2}({\vec{r}}_{m},{\vec{r}}_{c})}{\Delta D_{\text{max}}^{2}}}\right\},$$

where $r({\vec{r}}_{m},\;{\vec{r}}_{c})$ is the distance between the measured and calculated points; $\delta ({\vec{r}}_{m},\;{\vec{r}}_{c})$ is the dose difference between the same points; $\Delta D_{\max}$ is the dose‐difference criterion; and $\Delta d_{\max}$ is the distance‐to‐agreement (DTA) criterion. The regions where γ<1 correspond to locations where the experimental dose confirmed the computed dose within the acceptance criteria.

In a previous paper,^(10)^ a 1D array LA48 with high spatial resolution was used to determine the dose profiles for IMRT beams, and the passing criteria $\Delta D_{\max}=4\%$ and $\Delta d_{\max}=4\%$ mm for the TPS Plato Sunrise dose computation were selected so that Cilgam supplied more than 95% of the γ‐values less than 1. The same software was used in this work to compare the measured doses obtained by the 2D array and the computed dose by the TPS Plato.

### D. Treatment‐planning system

The Plato inverse treatment planning (ITP) module is contained in the Plato TPS version Sunrise 2.6.3 (Nucletron), which optimizes IMRT beams individually for each patient and tumor location. By means of absolute dose limits for overdosage of organs at risk (OARs) and for overdosage and underdosage of the tumor, the oncologist is able to control the optimization to a wide extent. Weight factors allow an additional ranking of these dose limits according to clinical importance. The beam setup parameters (gantry angle, energy) and fractionation schemes are defined in the Plato radiation therapy planning software (RTS) module (a conventional 3D treatment‐planning and calculation module). The RTS is implemented with dose profiles measured by a PTW thimble ion chamber model TM31002 (mentioned in section B). A natural diamond (model 60003)^(16)^ was used to improve the modeling of the penumbra regions for profiles of beam sizes ranging between $1\times 1\;{\text{cm}}^{2}$ and $3\times 3\;{\text{cm}}^{2}$. The implementation of the RTS for beams up to $2\times 2\;{\text{cm}}^{2}$ supplied an accuracy of the computed profiles of 3% in dose difference and 3 mm in DTA. The disagreement is essentially due to the modeling of the penumbra regions of the Plato TPS for small fields. This is the reason why, generally, segments less than $2\times 2\;{\text{cm}}^{2}$ are eliminated^(17)^ during the planning process. For beam sizes equal or greater than $3\times 3\;{\text{cm}}^{2}$, the accuracy was 2% in dose and 2 mm in DTA.^(15)^

**Figure 2:**
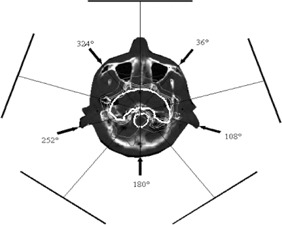
Tomography of a Rando head section. The concave target volume and a small OAR (circular in shape) are reported (white lines), together with some relative isodoses. The arrows show the direction of the five selected incident IMRT beams at 36°, 108°, 180°, 252°, and 324°; the five external lines are indicative of the “calculation plane” where the dose distributions have been computed.

To treat the head tumor shown in Fig. 2, five 6‐MV X‐ray beams located at the angles of 36°, 108°, 180°, 252°, and 324° were selected. The beam setup parameters defined in the RTS module were then passed to the ITP module. This module is comprised of an optimization, a sequencer, and a dose display module. The ITP calculation algorithm is a 3D single pencil beam convolution. The program takes into account the restrictions of the Elekta MLC (minimum distance between opposing leaves, transmission of the backup jaw, tongue, and groove). To start with, the user defines dose or dose‐volume constraints to the target and to the OARs by entering the optimization parameter table or by defining points for a desired dose‐volume histogram (DVH). During the optimization process, isodose lines are displayed on transverse and reformatted CT images, and DVH curves and fluence maps are updated. A good plan optimization was found using a total of 45 sequences. In particular, to obtain a dose of 1.8 Gy with five beams, the sequence number per beam ranged between 5 and 15, while the MUs per sequence ranged between 10 and 40.

After the dose optimization and the dose calculation are completed in ITP, the sequences and relative MUs are then sent to the RTS module, which gives the final dose calculation by using the more precise 3D pencil beam algorithm. The dose was calculated throughout every CT section with a 2‐mm dose grid resolution. The calculation plane was in a PMMA slab, 2 cm thick, at a depth of 1.3 cm (1.55 cm of water‐equivalent material), simulating the position of the 2D array at SDD=120 cm. The 20 cm distance from isocenter to calculation plane is ideal because all five calculation planes fit within the FOV area (48 cm in diameter) of the Aura Philips CT scanner used in this work. For each of the five treatment beams, the PMMA slab was drawn within the FOV with the Rando head sections.^(10)^

The 3D dose distribution contained in the TPS dose file was interpolated by the custom software Azabu to obtain the dose values on the calculation planes at different angular orientations (Fig. 2). The dose profiles along the lines reported in Fig. 2 were computed with a 2‐mm resolution, while the distances between the profiles parallel to such lines (along the longitudinal axis of the phantom) were 2.5 mm.

Before transferring the plan via DICOM‐RT for later delivery on the Elekta Precise LINAC, the five gantry angles were changed to 0° to allow the irradiation of the Rando head as described in the following section.

### E. Portal dose determinations

**Figure 3:**
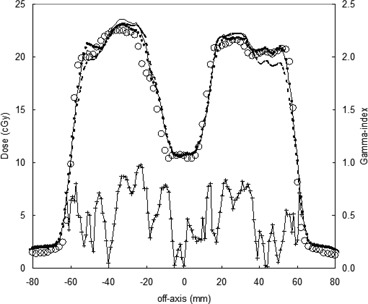
Comparison between a measured dose profile by an LA48 linear array along the principal axis, (O), and three computed dose profiles, the profile along the principal axis (continuous line) and other two at +2.5 mm (‐‐‐‐‐) and −2.5 mm (······) laterally. The γ‐index (—+—), computed with the acceptance criteria $\Delta D_{\max}=4\%$ and $\Delta D_{\max}=4$ mm, is reported on the right scale.

In a previous work,^(10)^ the PTW LA48 linear array, positioned at SDD=120 cm, was used to determine the acceptance criteria of the computed portal dose supplied by the TPS Plato. In the first step, the Rando female phantom was irradiated with antero‐posterior square fields. Every transmitted dose profile was determined in current mode acquisition with 1 mm spatial resolution, obtained using the automatic shift of the linear array along the latero‐lateral direction of the Rando phantom during the beam irradiation. The agreement between computed and measured portal dose profiles of square beams was quantified by Cilgam. The resulting percentage of points with γ<1 was greater than 95% $\left(P_{\gamma <1}>95\%\right)$, when the acceptance criteria $\Delta D_{\max}=3\%$ and $\Delta d_{\max}=3$ mm were selected. In a second step, the LA48 array was used to test the accuracy of the computed portal dose for IMRT irradiation. The Rando head sections were locked between two PMMA slabs with a mechanism that allowed the phantom head to be rotated around the isocenter point and five IMRT beams were delivered, maintaining the LINAC gantry at 0° (Fig. 1). The dose distributions were computed by the TPS Plato and extracted by Azabu for comparison with the experimental data obtained by the LA48. In this case, the LA48 worked in integral mode acquisition, and 50 iterations of the IMRT beam were used to obtain, at 120 cm from the source, a portal dose matrix with a 2‐mm spatial resolution along the profile and 1 cm between the profiles. Figure 3 shows the comparison between a measured dose profile along the principal axis (which intercepts the beam central axis) and three computed dose profiles. The first computed profile is along the principal axis, and the other two are at ±2.5 mm laterally. Using Cilgam for the γ‐index analysis, a percentage $P_{\gamma <1}>95\%$, with the acceptance criteria $\Delta D_{\max}=4\%$ and $\Delta d_{\max}=4$ mm, was obtained for the five IMRT beams.^(10)^

It is interesting to note that using the LA48 device in water phantom for pretreatment verification of the computed IMRT plan, a $P_{\gamma <1}>95\%$ was obtained with acceptance criteria of 3% in dose difference and 3 mm in DTA. These results are in agreement with others reported in the literature,^(18)^ where it is emphasized that the disagreements are generally due to the level of accuracy of the calculation in high‐dose gradient regions for small segments.

In this work, the same five IMRT beams used in the previous work(^10^) were used, and the computed IMRT portal dose distributions were compared with the measured portal doses obtained by the 2D array. All the transmitted dose measurements below the Rando phantom were carried out with the gantry at 0° and $330\;\text{MU}\;\min^{-1}$, rotating the phantom head around the isocenter point (Fig. 1). Using the five IMRT beams, a dose of 1.8 Gy at the isocenter was delivered. The total time to deliver the plan to the phantom was approximately 15 min. Two minutes were required for irradiation, and the rest was used to modify sequences and phantom angles between beams.

## III. RESULTS

### A. 2D array calibration

**Figure 4:**
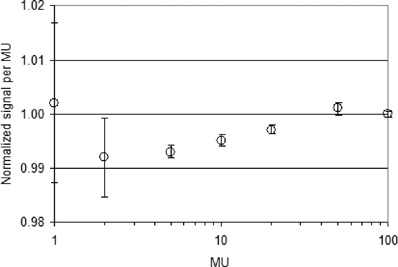
Signal per MU normalized to 100 MUs obtained by the central ion chamber of the 2D array when the MU changed between 1 and 100. The bars indicate measured reproducibility (1σ).

The dose measurements carried out in this work for the 2D array commissioning show that the signal reproducibility was estimated to be better than 0.1% (1σ) for a number of MUs≥5 (≥0.05 Gy) and better than 0.5% (1σ) for MUs≥2 (≥0.02 Gy). Figure 4 shows, for the central axis ion chamber of the 2D array, the trend of the signal per MU (normalized at 100 MUs) obtained when the MUs changed between 1 and 100. This ratio was within 0.5% for MUs greater than 10. The bars show the reproducibility (1σ) of the normalized signal.

The 2D array does not allow correction of ion recombination by two voltage techniques. However, ratios between the signals obtained by the central 2D array ion chamber and the reference PTW thimble ion chamber were constant within the experimental reproducibility (0.2% 1σ) in the range of dose rate between $0.1\;\text{Gy}\;\min^{-1}$ and $5\;\text{Gy}\;\min^{-1}$. This means that the correction factor for the ion recombination of the 2D array ion chambers was of the same order as that of the PTW reference chamber (1.002) and could be negligible for our purposes.

The linear fit of the 2D array central chamber signal, *M*, corrected for standard pressure and temperature as a function of the dose value, *D*, was allowed to obtain a calibration factor N=0.2024 Gy/nC and a regression coefficient of 0.999. This calibration factor was initially associated with all 729 ion chambers. The comparisons between the dose profiles for a $30\times 30\;{\text{cm}}^{2}$ beam, obtained by the reference ion chamber and the 2D array positioned at the buildup of water and PMMA phantoms, respectively, allowed the correction of the calibration factors. Due to the small differences in size of the chamber volumes, the variations between these factors, normalized at the central ion chamber, were within ±1%.

**Figure 5:**
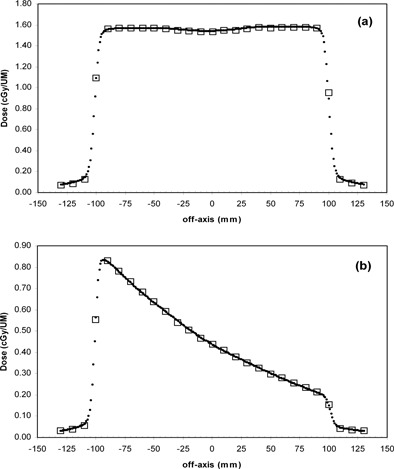
Comparison between dose profiles of the (a) open and (b) wedged fields, $20\times 20\;{\text{cm}}^{2}$ in size, obtained with the LA48 linear array (•) and the 2D array (□).

The dosimetric data obtained in standard phantoms by the 2D array, compared with the dose profiles obtained by the LA48 (ion chambers with 8 mm^3^ in volume), showed discrepancies with $\Delta D_{\max}=2\%$ and $\Delta d_{\max}=2$ mm. Figure 5 shows an example of the comparison between profiles of open and wedged fields measured with the 2D array and those obtained by the LA48 linear array. These data were in good agreement with those obtained by Spezi et al.,^(13)^ who observed a maximum discrepancy of $\Delta D_{\max}=3\%$ for the IM beam profiles obtained by the 2D array and a pinpoint ion chamber with a volume of 16 mm^3^.

The deviations between the $S_{\text{cp}}$ values obtained by the 2D array and the reference PTW ion chamber were less than 1%, justifying the negligible effect of the neighboring ion chambers.

Comparing these results with those obtained by the dose characterization of MapCHECK,^(18)^ it is possible to conclude that the PTW 2D array presents minor dose rate dependence as compared to 2% or 3% for *n*‐type or *p*‐type diodes. However, in a recent paper(^19^) a comparison of the dosimetric characteristics of the 2D array PTW, MapCHECK (Sun Nuclear, Melbourn, FL), and the more recent PIX (Pixel ionization chamber prototype)(^20^) shows that these 2D arrays are useful tools for the quality assurance and verification of the IMRT plans in the pretreatment step.

### B. Portal dose determinations

**Figure 6:**
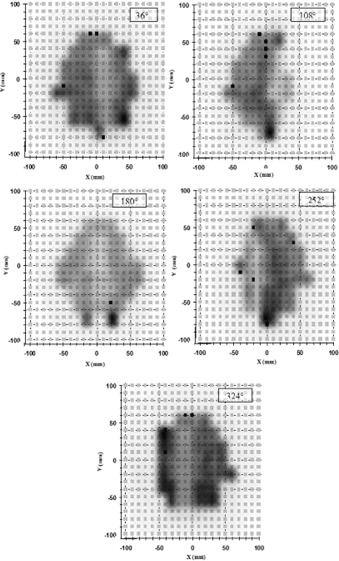
Zoom of the 2D array panels (21×21 chambers instead of the 729) with the shadowed areas showing the modulation of the five IMRT computed beams obtained with 6‐MV X‐ray beams. The closed, square symbols represent the ion chambers where the resulting γ‐index was greater than 1 for acceptance criteria $\Delta D_{\max}=4\%$ and $\Delta d_{\max}=4$ mm.

Figure 6 shows a zoom of the 2D array panels (21 × 21 ion chambers) as presented by VeriSoft, with the shadowed areas showing the virtual film of the five intensity beams. The closed square symbols represent the ion chambers where the resulting γ‐index was greater than 1, for acceptance criteria $\Delta D_{\max}=4\%$ and $\Delta d_{\max}=4$ mm as computed by Cilgam. With accurate Rando phantom centering, the number of the closed symbols resulted less than 5% for all five beams. The measurements were repeated five times obtaining the same result.

**Figure 7:**
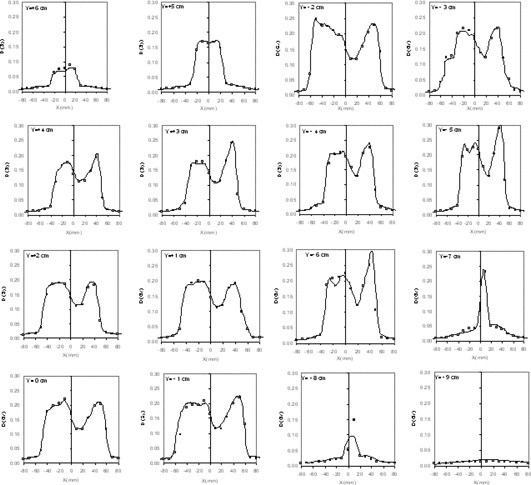
Dose profiles computed by the TPS (continuous lines) at different *Y* distances, along the lines of ion chambers of the 2D array (□) for the IMRT beam with incidence angle of 36°. The closed square symbols represent the points where the differences between computed and measured doses are outside the acceptance criteria.

For the IMRT beam located at 36°, Fig. 7 reports 16 dose profiles computed by the TPS on the calculation plane, to compare them with the measured portal dose by the 2D array. Two hundred and seventy‐two ion chambers received a dose of at least 5% of the maximum dose value (0.3 Gy) and were considered in this analysis. Each of the five beams was irradiated between 150 and 280 ion chambers with at least this threshold value.

**Figure 8:**
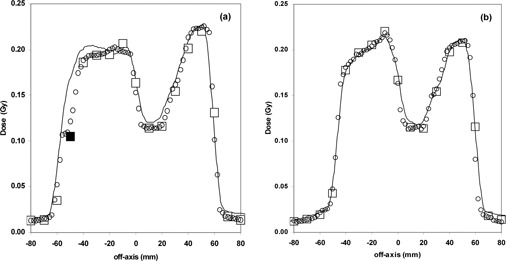
Comparison between measured portal dose profiles for the IMRT beam at 36° at (a) Y=−1 cm and (b) Y=0 cm obtained by an LA48 (○) and a 2D array (□), with the profile computed by the Plato (continuous line). The closed symbol is relative to ion chamber of the 2D array where the γ‐index is greater than 1.

Figure 8 reports, for the beam at 36°, an example of the comparison between measured portal dose profiles at Y=−1 cm and Y=0 cm obtained by the 1D‐array LA48 and the 2D array, with the profile computed by Plato. The agreement between the dose profiles obtained by the linear detector and 2D detector determined by Cilgam is within $\Delta D_{\max}=2\%$ and $\Delta d_{\max}=2$ mm. The closed symbol is relative to the ion chamber of the 2D array where the γ‐index is greater than 1. Considering that the acceptance criteria of the TPS portal dose calculation were estimated to be 4% and 4 mm, the loss of agreement between the dose calculation and the 2D array measurements in less than 5% of the chambers (Fig. 6) is a good result, considering that about 1000 dose points were checked.

**Figure 9:**
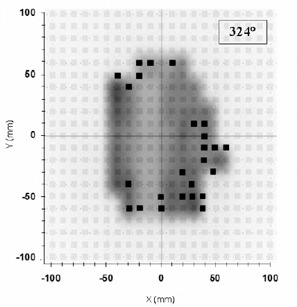
Zoom of the 2D array panels (21×21 chambers instead of the 729) with the shadowed areas showing the modulation of the IMRT computed beam obtained with a 6‐MV X‐ray beam at 324°. The measurement was carried out with the beam collimators misaligned by 5° around the central axis. The closed square symbols (about 15%) represent the ion chambers where the γ‐index resulted greater than 1 for acceptance criteria of $\Delta D_{\max}=4\%$ and $\Delta d_{\max}=4$ mm.

Six 5‐mm shifts of the isocenter point were simulated moving the Rando phantom (±5 mm) along the three principal axes. The percentage $P_{\gamma <1}$ ranged between 85% and 90%, maintaining the acceptance criteria of 4% and 4 mm. For the beam at 324°, Fig. 9 presents the zoom of the 2D array panel as presented by VeriSoft, when the beam collimators were misaligned by a 5° rotation around the central axis. The percentage $P_{\gamma <1}$ decreased to 85%, and the result can be compared with that reported in Fig. 6 for the same beam.

## IV. DISCUSSION AND CONCLUSIONS

A dosimetric quality assurance (QA) program in complex radiotherapy treatment plans such as IMRT requires advanced technology. However, for IMRT, besides a complete program for the QA of the LINAC, the simulator, the TPS, and independent checks of radiotherapy treatment parameters (distances, field size, etc.), a pretreatment control of the computed dose in standard phantom for every beam is mandatory.

In many centers, pretreatment verification of the dose calculation for the beams selected for patient therapy is carried out by 2D arrays as the PTW here reported. However, this verification requires a lot of work before treatment, which interrupts the activity of the radiotherapy department for 15 min or 30 min, depending on the number of fields. To avoid such interruptions, this verification is not generally repeated in the following fractions of the therapy. It is our opinion that the first pretreatment verification is mandatory for the control of dose computation, while it could also be useful to check the following fractions of the therapy to verify the constancy of (1) the correct dose delivery and (2) correct patient positioning.

EPIDs are probably the most promising devices for IMRT verification and *in vivo* dosimetry.^(^ ^8^ ^,^ ^21^ ^,^ ^22^ ^)^ These detectors can provide high resolution and highly efficient planar dose maps. However, further developments are underway for the deployment of this technique into routine clinical practice.^(^ ^23^ ^–^ ^25^ ^)^ In the meantime, we think that 2D online dose detectors could be used together with EPIDs for complete *in vivo* QA purposes.

The PTW 2D array here examined is a practical detector for radiotherapy verification even if the ion chamber spacing of 1 cm results in a limited sampling of the radiation beam. However, this limitation does not seem to affect its possible use to accurately check several dose points. The dose calibration for the 2D array is easy and stable. For a small number of MUs (≥10) the step‐and‐shoot sequences of our IMRT offer a high level of dosimetric accuracy (Fig. 4). Moreover, this array seems to be a water‐equivalent detector.

The 2D array was simulated in a TPS for the IMRT portal dose computation using a slab of water‐equivalent material. This way, custom software was realized to extract from the TPS the maps of portal doses on specific planes in the FOV.

In irradiation of a Rando phantom with step‐and‐shoot IMRT beams, and measuring portal doses with a 1D array with high spatial resolution, we have determined that the TPS Plato Sunrise supplies a dose computation with a $P_{\gamma <1}>95\%$ of portal dose points with acceptance criteria of $\Delta D_{\max}=4\%$ and $\Delta d_{\max}=4$ mm. Figure 6 shows the comparison between the computed and measured portal dose values by the 2D array. The number of chambers that found a dose computation within the acceptance criteria is greater than 95%. In other words, the good agreement observed between the measured dose profiles obtained in standard phantoms by the 1D array LA48 and the 2D array ($\Delta D_{\max}=2\%$ and $\Delta d_{\max}=2$ mm) justifies the 2D array's $P_{\gamma <1}>95\%$. We think that the discrepancies are due both to the accuracy level of the portal dose calculation as well as the grid (2.5 mm) used by Azabu in the sampling of the dose points between the different portal dose profiles.

In conclusion, even if the 2D array presents a limited sampling capability, it is our opinion that the observation of about 1000 points (200 per beam) is enough for a detailed evaluation of the portal dose reproducibility in many fractions of the therapy. This kind of verification can be carried out during the treatment, positioning the 2D array in a particular jig that can follow the rotation of the portal vision.

The effect of 5‐mm shifts of the Rando head allows one to estimate a decrease of $P_{\gamma <1}$ to 85% of points inside the acceptance criteria of $\Delta D_{\max}=4\%$ and $\Delta d_{\max}=4$ mm. This result can be useful for the *in vivo* QA. Moreover, the procedure allows the observation of an incorrect angular position of the multileaf collimator. Figure 9 shows a drop of $P_{\gamma <1}$, to 85% when an error of 5° in the rotation of the beam collimators was simulated.

In conclusion, the procedure here tested, based on step‐and‐shoot IMRT irradiation of the Rando head, can be used for head and neck tumors where the computation of the dose is well within the FOV area of the CT scanner. We intend to use it for the IMRT *in vivo* treatments to supply a contribution to the selection of the percentage of points $P_{\gamma <1}$ and the passing criteria significant for the judgment of the reproducibility of therapy fractions.

## ACKNOWLEDGMENTS

We are grateful to Nuclital s.r.l., Dr. A. Bursi, and Dr. G. Alberta for their technical assistance.

## Supporting information

Supplementary Material
