# Supplementary material for: Comparison of measured and computed portal dose for IMRT treatment
Source: J Appl Clin Med Phys. 2006 Aug 24;7(3):65–79. doi: 10.1120/jacmp.v7i3.2281 (PMC5722422; doi:10.1120/jacmp.v7i3.2281)
Supplement: Supplementary file 1 — Supplementary Material [file ACM2-7-65-s001.doc]

Reply to the referee’s comments.

Thank for your comments that we have taken into great consideration.

General Comments.

We agree that the sentence that we have used frequently in the text: ‘the lack of spatial resolution of the 2D-array does not prevent to accurately detect the IMRT dose distributions’ seems to claim that the 2D-array is fully addressed for IMRT fields. However, this device is used today for pre-treatment tests of conformal and IM beams giving in the last case good dosimetric results without being time consuming.

About the 2D-array dose spatial resolution as reported in the text, the film requires extreme cure in the processing (generally the film is oversensitive to low-energy photons which may be important in penumbra regions) and as in many Centers (as ours Centre) the chemical processing of film is absent.

The pin-point ion-chamber presents a diameter of 3mm and 3mm in length (18mm^3^ in volume) and requires many hours of work for IM beam analysis .These are the reasons why we selected the LA48 (that contains 47 liquid ion-chambers 8mm spaced apart, 4x4mm^2^ in size, 0.5mm thick, 8mm^3^ in volume) as reference dosimeter.

However, we agree with your suggestion and now we are reporting in the paper the steps as follows:

1. the 1D-array was tested with a diamond detector, (ref 10);
2. using the Cilgam analysis, the agreement of the 2D-array with the 1D-array resulted within ΔD_max_=2% and Δd_max_=2mm.

In figure 8 we intended to show that, within the above dosimetric agreement between the two arrays, both the devices were in disagreement with the computation. Now figure 8 b) reports the comparison of the same beam for Y=0 where the agreement is more stringent. We think that the discrepancies between the 2D measurements and the computations can be due both to the curacy level of the TPS computed portal dose, as well as to the sampling (2.5 mm)used by Azabu software between the portal dose profiles.

In conclusion, we think that the “value of the 2D-array as a dosimetric Q.A. tool for IMRT is in its feasibility to obtain about 1 thousand dose points during many fractions of the head-neck tumors (where the IMRT is very indicated) using the FOV area of the CT scanner. The results obtained for the first patient recently checked with the 2D-array show a good reproducibility of the measurements and we intend to present these results when we have an adequate number of patients.

We apologize for our English and we have submitted the paper to an English expert.

## Specific Comments (at your numbers)

1. Para. 3: We hope we have understood the comments. In literature acceptance criteria for the TPS Plato computation of the portal dose distribution are not reported. However, the sentence has been changed. We have reported a comparison between measured and computed portal dose values reported in a previous paper.

Para. 4 was rephrased

1. The ‘correct leaf position’ is now mentioned.
2. The reasons why we have selected the LA48 ion chambers (with 8mm^3^ in volume) as reference detector, are now better specified. Indeed, in the previous work (ref.10), the dosimetry of this 1D-array was compared with a PTW diamond detector.

In the text of the paper it is reported that, for field of dimensions above 2x2 cm^2^ we obtained dosimetric discrepancies between the diamond detector and the LA48 linear array equal to:

ΔD_max_ =1.5% and Δd_max_=1mm confirming other results (ref. 11).

The comparison between 1D-array and 2D-array measurements carried out in phantoms allows to define discrepancies well within ΔD_max_ =2% and Δd_max_=2mm.

6) This level of accuracy for fields of small size is also reported by other authors, and this is due to the modeling of penumbra regions in the planning systems for very small segments (ref.17) (This is the reason why segments with small sizes (<2x2cm^2^) are generally not taken into consideration when planning). However, in the previous test the percentage of 4% was a misspelling.

1. Page 9. We agree that the comparison is between two detectors with the same dimensions. However, as reported in the previous point 3), the LA48 was commissioned with a diamond detector.
2. Page 10, para. 1. In the text we have reported that the discrepancy between the LA48 and the 2D-array is within ΔD_max_ =2% and Δd_max_=2mm, and the discrepancies with the computation are the same (figure 8a). In the figure 8b is reported the agreement for Y=0cm for the same beam.

Page 10, para. 2. The sentence has now been rewritten more clearly.

Page 10, para. 4. The sentence was erased.

1. Page 11. See points 3 and 8. In the discussion section we have underlined that the acceptance criteria of the Plato computation , ΔD_max_=4% and Δd_max_=4mm for a P_γ<1_>95% of the portal dose points examined (with a Rando head) , are greater than the discrepancies of the 2D-array as respect to the 1D-array (this last commissioned with a diamond detector). This can justify the result of the a P_γ<1_>95% between 2D and dose computation.
2. Page 11, para. 2. The sentence is now clearer. In the previous paper (ref. 10) we examined 3 TPSs and the portal dose calculation was more correct with the PLATO TPS. In general, for distances of the EPID, the computation of the portal dose is not accurate. For tumors of the head-neck, where it is possible to use a 2D-array near the patient, the computation of the Plato seems to be accurate enough. We do not know about works in this field, in particular where distances less than the EPID distances have been examined.
